# Review on practical photoacoustic microscopy

**DOI:** 10.1016/j.pacs.2019.100141

**Published:** 2019-08-09

**Authors:** Seungwan Jeon, Jongbeom Kim, Donghyun Lee, Jin Woo Baik, Chulhong Kim

**Affiliations:** Department of Creative IT Engineering, Pohang University of Science and Technology (POSTECH), Pohang, 37673, Republic of Korea

**Keywords:** Optoacoustics, Resolution, SNR, Fast scanning

## Abstract

Photoacoustic imaging (PAI) has many interesting advantages, such as deep imaging depth, high image resolution, and high contrast to intrinsic and extrinsic chromophores, enabling morphological, functional, and molecular imaging of living subjects. Photoacoustic microscopy (PAM) is one form of the PAI inheriting its characteristics and is useful in both preclinical and clinical research. Over the years, PAM systems have been evolved in several forms and each form has its relative advantages and disadvantages. Thus, to maximize the benefits of PAM for a specific application, it is important to configure the PAM system optimally by targeting a specific application. In this review, we provide practical methods for implementing a PAM system to improve the resolution, signal-to-noise ratio (SNR), and imaging speed. In addition, we review the preclinical and the clinical applications of PAM and discuss the current challenges and the scope for future developments.

## 1 Introduction

Photoacoustic (PA) effect is a phenomenon where light energy is converted into ultrasound (US) energy [1]. In the PA effect, a sample irradiated by a short pulsed light thermally expands and generates US waves instantaneously. The initial local pressure of the US waves generated can be expressed as [2,3].

(1) $$p_{0}\left(\vec{r}\right)=\eta \text{Γ}\mu_{a}F\left(\vec{r}\right),$$

where η is the heat conversion efficiency, Γ is the Grueneisen parameter that increases with local temperature, $\mu_{a}$ is the absorption coefficient, and $F(\vec{r})$ represents the optical fluence. Based on this PA effect, a biomedical imaging technique called photoacoustic imaging (PAI) reconstructs PA images with optical contrast and US resolution [[4], [5], [6]]. Thus, at the location where the light is well absorbed and the temperature is high, the PA images exhibit the highest signal-to-noise ratio (SNR). Since the PAI is based on the PA effect, it provides both structural and functional information (e.g., hemoglobin oxygen saturation (sO_2_), hemodynamics, metabolism) of blood vessels without any external contrast agent [[7], [8], [9], [10], [11]]. These features are very important in the biomedical studies since the morphological and functional abnormalities of the vessels may be the early indicators of various diseases [12,13]. The PAI is also capable of performing molecular imaging, such as in cancer targeting using light-absorbing exogenous contrast agents [[14], [15], [16]]. More importantly, PAI has the advantage of being safe since there is no ionizing radiation involved in most of the cases.

Depending on the system configuration and the imaging depth, the resolution of PAI can range from nanometer to millimeter [[17], [18], [19]]. In photoacoustic microscopy (PAM), which inherits the characteristics of PAI, micron-scale spatial resolution can be achieved and is used for *in vivo* microvascular imaging. PAM has several advantages over the conventional optical microscopy such as confocal, two-photon microscopy, or optical coherence tomography (OCT). First, PAM has a deeper imaging depth beyond the optical diffusion limit of 1 mm since it uses the US to form the image that is less scattered in biological tissue than light. Second, PAM can provide the structural and functional information of each microvessel with high sensitivity. Third, PAM does not need optical sectioning to obtain a 3D volume image.

The PAM systems have been developed in several forms for many years and are used in many applications such as vascular biology [20,21], histology [22,23], oncology [24], neuroscience [25], and ophthalmology [26,27]. In this review, we present representative PAM system implementation methods and recent advances in PAM techniques. Furthermore, we will discuss the potential preclinical and clinical application, and the remaining challenges of PAM.

## 2 Major implementation categories of PAM

The PAM systems are generally classified into two categories based upon the type of foci, i.e., the acoustic and the optical foci, used to determine the lateral resolution. Namely, acoustic-resolution PAM (AR-PAM) and optical-resolution PAM (OR-PAM). The maximum penetration depth of each type is different and both generate high-resolution images using optical ballistic regime (in OR-PAM) and optical diffusive regime (in AR-PAM). The following paragraph describes several representative system configurations and their features. Researchers need to understand their differences and choose the right system configuration. If necessary, it can be fused with other PAM configuration such as a switchable OR and AR-PAM system [28,29] or a double-illumination PAM system [30].

**Fig. 1:**
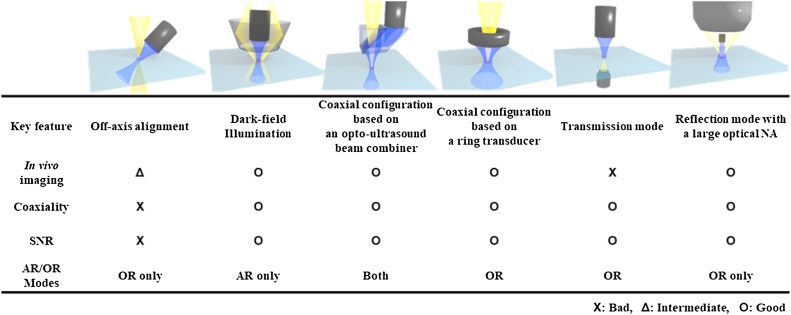
Features of various PAM implementation types. PAM, photoacoustic microscopy; NA, numerical aperture; AR, acoustic resolution; and OR, optical resolution.

The PA signals are generated at the point where the optical and acoustic foci overlap. Thus, it is recommended to align these two beams coaxially to maximize the PAM image sensitivity. Many technologies have been developed to achieve this coaxial configuration between the US and the optical beams in the PAM systems. Fig. 1 shows the representative methods for implementing PAM system. One of the simplest ways is to place an acoustic detector and a light source off the axis to each other [31,32]. The PAM system designed with this configuration would use a fixed unfocused acoustic area. In this system, the detection sensitivity and field of view (FOV) are limited and is difficult to use it in AR-PAM mode. In addition, the misalignment between the excitation light and the emitted acoustic beams limit the axial resolution [31]. This PAM type is mainly used for imaging flat cell samples or imaging very narrow areas of small animals [33]. The second type is dark-field confocal PAM. In this system, a conical lens is used to make an annular light beam pattern that bypasses the US transducer. After passing through an optical condenser, the light is focused on the sample [34,35]. The US transducer detects the generated US waves. In this case, the coaxial configuration between the excitation light and the emitted US can result in the high SNR. The only downside of this approach is that only AR-mode can be implemented since it is difficult to focus the light tightly. This type is commonly used for the whole body, internal organs, and for deeper vessels imaging [36]. The third method uses an opto-acoustic beam combiner to combine light and US coaxially. This type of PAM is based on the opto-acoustic beam combiner and can focus the light tightly, achieving the lateral resolution less than 5 μm. This system can also achieve a penetration depth of approximately 1.2 mm in live animals [37,38]. However, the beam combiner could limit the use of high optical numerical aperture (NA) and can cause acoustic energy loss of about 30% due to the acoustic impedance mismatch. This beam-combiner-based PAM is widely used in *in vivo* microvessel imaging in OR-PAM mode, and can also be used in AR-PAM mode using loosely focused light [39]. Because of this compatibility, a switchable OR and AR-PAM system can be implemented by changing a few optical components [40] or using an optical fiber bundle [41]. The fourth method, PAM based on a ring transducer, uses a US transducer with a hole in which light can pass through. This kind of PAM can achieve the resolution and SNR as high as that of the third method, though the hole in the US transducer results in about 10% energy loss and also restricts the optical NA. The ring-transducer-based PAM is usually used for applications similar to the beam-combiner-based PAM in OR-PAM mode. [42]. The fourth transmission-mode PAM method detects the US signal from the other side of the light source. This method is useful in cell imaging in OR-PAM mode since the optical NA can be maximized above 1.0 [43,44]. However, unlike the previous methods, this type has the disadvantage that it cannot be used for *in vivo* imaging because of the way the system is configured. The last method can be an alternative to solve the problem of the transmission mode [[45], [46], [47]]. This method uses a very high NA optical lens and a tiny transducer just below the optical lens. Although the focal length is quite short due to the high NA, *in vivo* imaging is available with the subwavelength resolution.

## 3 Spatial resolution

The lateral resolution of AR-PAM, ${LR}_{AR-PAM}$, depends on the acoustic properties as follows:

(2) $${LR}_{AR-PAM}=0.71\frac{\lambda_{a}}{{NA}_{a}}=0.71\frac{v}{{NA}_{a}\times f_{c}},$$

where $\lambda_{a}$ is the center wavelength of the detected PA signal, ${NA}_{a}$ is the acoustic NA, v is the speed of sound, which is typically fixed to 1540 m/s for biological tissue, and $f_{c}$ is the center frequency of the PA signals. Thus, the lateral resolution of AR-PAM can be improved by increasing the center frequency and NA of the US transducer. However, the high NA can significantly deteriorate the lateral resolution in the out-of-focus region. We discuss this phenomenon and its solution in detail in Section 4.4. The increased center frequency also results in increased acoustic attenuation, limiting the imaging depth. Therefore, when designing an AR-PAM system, it is necessary to carefully consider the tradeoff between the imaging depth and the lateral resolution.

In OR-PAM, the lateral resolution is determined by the optical focal spot size [43],

(3) $${LR}_{OR-PAM}=0.51\frac{\lambda_{o}}{{NA}_{o}},$$

where $\lambda_{o}$ denotes the optical wavelength and ${NA}_{o}$ denotes the optical NA. The OR-PAM achieves high lateral resolution with a tight optical focus but the imaging depth is restricted to the mean optical path length (about ˜1 mm for biological tissue) [48].

As for the axial resolution, $\;{AR}_{PAM}$, there is no difference between AR-PAM and OR-PAM because their axial resolution is mainly determined by the acoustic bandwidth as follows:

(4) $${AR}_{OR-/AR-PAM}=0.88\frac{v}{\text{Δ}f_{c}},$$

where $\text{Δ}f_{c}$ is the bandwidth of the PA signal. The PA signal has a very wide bandwidth ranging from 1 MHz to 100 MHz [49] and this range is much wider than the detectable bandwidth of the conventional piezoelectric transducer (PZT). Thus, it is safe to assume that the bandwidth of the received PA signals is equal to the bandwidth of the detector.

**Fig. 2:**
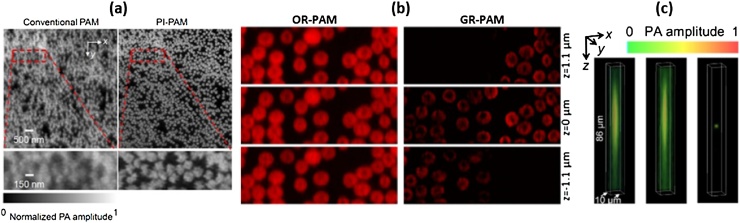
(a) Hemoglobin imaged by conventional OR-PAM (left) and PI-PAM (b) Optically sectioned hemoglobin images at different depth acquired by conventional OR-PAM (left) and GR-PAM (right) (c) Volumetrically rendered 0.5-μm beads images at left (left), right (middle), and dual (right) views. PI-PAM, photoimprint PAM; GR-PAM, Grueneisen relaxation PAM; OR-PAM, optical-resolution PAM; and PAM, Photoacoustic microscopy. Reproduced with permission from Ref. [50,52,54].

Recently, several PAM systems with improved spatial resolution beyond those defined above have been introduced. In 2014, Yao et al. introduced label-free subdiffraction OR-PAM, called photoimprint PAM (PI-PAM), based on a double excitation process (Fig. 2a) [50]. When a sample is illuminated by an excitation beam, absorbers in the sample not only produce photoacoustic waves but are also heterogeneously photobleached. When the second laser pulse excites the photobleached area within a few microseconds, the PA signal decreases. Mainly, the photobleaching effect is more dominant in the center than the periphery. As a result, the differential image between the PAM images before and after photobleaching has a smaller point-spread-function (PSF) than the conventional OR-PAM image. They imaged hemoglobins using an oil-immersion objective lens of 1.4 NA at 532 nm. They then demonstrated that the PI-PAM could improve the lateral resolution from 200 nm to 120 nm. In a similar way, Yao et al. demonstrated another subdiffraction PAM using a reversibly switchable bacterial phytochrome called BphP1 [51]. BphP1 exists in two states, ON and OFF. BphP1 in ON and OFF states produces a PA signal when illuminated at wavelengths of 780 nm and 630 nm, respectively, and is switched into the opposite state. When successive laser pulses illuminate a spot, the PA signal at the center of the spot continuously decreases more rapidly as compared with the periphery. Using this principle, they improved the lateral resolution from 287 nm to 141 nm and the axial resolution from 29.5 μm to 410 nm.

Wang et al. developed Grueneisen relaxation PAM (GR-PAM) to improve the spatial resolution by exploiting the nonlinearity in PA signal generation (Fig. 2b) [52]. In general, the amplitude of the generated PA signal is proportional to the optical fluence, as described in Eq. (1). However, the differential signal between the PA signals before and after preheating, refer to as GR signal, is nonlinearly proportional to the optical fluence due to the Grueneisen parameter. In particular, as an absorber is axially away from the optical focus, the GR signal rapidly decreases compared to the conventional PA signal. Using this effect, Wang et al. imaged hemoglobins with the improved axial resolution from 45 μm to 2.3 μm. Similarly, Liu et al. also developed a Grueneisen relaxation ultraviolet PAM (GRUV-PAM) and demonstrated label-free nuclear imaging with the axial resolution of 15 μm [53].

Dual-view OR-PAM, developed by Cai et al. in 2018, can also improve the spatial resolution (Fig. 2c) [54]. Cai et al. split a laser beam into two beams and concentrated them on a spot in perpendicular directions to each other. They acquired two view images by imaging the samples using the different illumination individually. Then they applied a double-view Richardson-Lucy deconvolution [55] to these two view images. As a result, the lateral and the axial resolution, which were originally 3.0 μm and 37 μm, respectively, were isotropically improved to 1.8 μm in the x–z plane.

## 4 Image enhancement in out-of-focus regions

Deep imaging depth and high sensitivity to blood vessels are great advantages of PAM in *in vivo* imaging, especially when compared to other microscopy techniques. Using near-infrared (NIR) light can extend the maximum imaging depth because biological tissues pose relatively less optical attenuation in this wavelength range [56]. Particularly, the second NIR window (NIR II; ˜1000 nm – 1350 nm) gains great attention because hemoglobin absorption is much lower and tissue scattering is much less compared to those in the first NIR window (NIR I; ˜700 nm – 950 nm) [57]. Further, according to the American National Standards Institute (ANSI), the maximum permissible energy for skin allowed in the NIR-II window is higher than the VIS and NIR-I optical ranges [58,59]. Therefore, the penetration depth of PAM can likewise increase in this range. However, the resolution of the PAM system remains good only at the depth of focus but significantly degrades elsewhere. Thus, many methods have been proposed to extend the depth of field (DoF) while maintaining the lateral resolution and the methods are mainly classified based upon the PAM modes. In a Gaussian beam-based OR-PAM mode, there is a trade-off between the lateral resolution and the DoF because high optical NA cannot provide long DoF. Here, we introduce a Bessel beam, focus-shifting lens, and the single pixel imaging technique for the OR-PAM mode, and synthetic aperture focusing techniques (SAFTs) for the AR-PAM mode.

### 4.1 Bessel beam (OR-PAM)

**Fig. 3:**
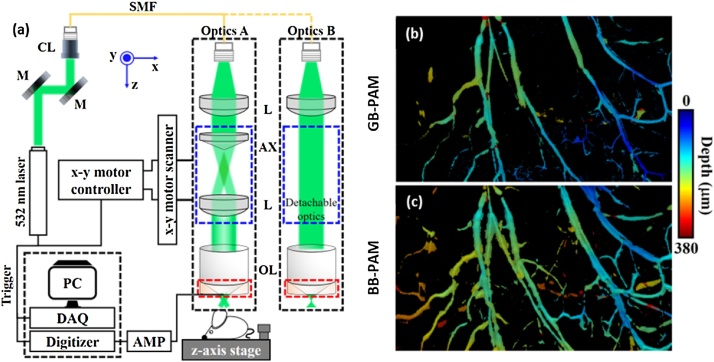
(a) System schematic of BB-PAM. (b) *in vivo* mouse ear MAP images of GB-PAM (top) and BB-PAM (bottom). PAM, photoacoustic microscopy; BB-PAM, Bessel beam PAM; GB-PAM; Gaussian beam PAM; L, lens; AX, axicon lens; OL, objective lens; M, mirror; CL, collimator; SMF, single mode fiber; and AMP, amplifier. Reproduced with permission from Ref. [47].

Bessel beam, generated by an axicon lens, has longer DoF than Gaussian beams and maintains acceptable lateral resolution over a longer depth range. Thus, this can reduce image degradation in the out-of-focus regions. Fig. 3(a) shows the schematic of the Bessel-beam PAM (BB-PAM) system developed by Park et al. [47]. This BB-PAM system can be easily modified to a Gaussian-beam PAM (GB-PAM) system by replacing a set of the optical system (blue boxes in Fig. 3(a)). Fig. 3(b–c) shows that the BB-PAM has longer DoF than the GB-PAM.

The main drawback of the BB-PAM is that it has strong sidelobes. Using blind deconvolution, an image processing technique, is one of the ways to overcome this problem. In blind deconvolution, the sidelobes of BB-PAM are corrected by estimating the optimal PSF of the system. Jiang et al. [60] and Park et al. [47] both used a blind deconvolution technique to reduce sidelobes in BB-PAM. Jiang et al. reported the BB-PAM’s DoF to be 483 μm, about 7.4 times longer than that of GB-PAM (65 μm), and the lateral resolution to be about 1.7 μm for both systems. Similarly, Park et al. successfully extended the DoF (229 μm) by about 7 times longer than GB-PAM (33 μm) and achieved sub-wavelength resolution (300 nm) using a high NA objective lens (NA: 1.0).

Another strategy to reduce the sidelobes is to use the Grueneisen relaxation effect [61]. The optical fluence of the Bessel beam is proportional to the square of the zero-order Bessel function of the first kind, ${J_{0}}^{2}$, and the sidelobes are derived from this ${J_{0}}^{2}$. If the sample is imaged immediately after being preheated by a laser pulse, then the PA signal increases according to the Grueneisen relaxation effect (refer to Eq. (1)). At this time, the difference between the PA signals with and without preheating is proportional to ${J_{0}}^{4}$, thus reducing the sidelobe amplitude. Using this technique, Shi et al. obtained a BB-PAM image with the reduced sidelobes and the measured lateral resolution and DoF were 6.1 μm and 1600 μm, respectively.

### 4.2 Focus-shifting lens (OR-PAM)

The defocused image can be improved by moving the Gaussian beam’s focus in the axial direction to compensate for the short DoF. Several researchers have addressed the short DoF problem by shifting the optical focus mechanically [62], but those methods restricted the volumetric imaging speed. This volumetric speed problem can be addressed by using a lens that can quickly adjust to the optical focal length.

**Fig. 4:**
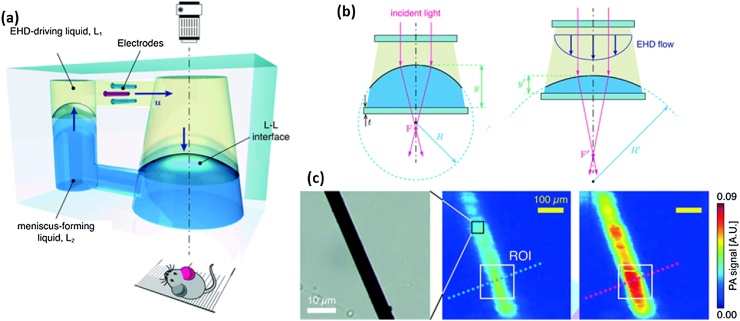
(a) Schematic of the DOL. When applying an electric field to the electrodes, the liquid interface, L-L, is modulated. (b) Focal length shifting according to the EHD flow. (c) A wide-field optical image of a 7-μm carbon fiber (left) and its PA MAP images before (middle) and after (right) adjusting the focal length. DOL, dielectro-optofluidic lens; EHD, Electrohydrodynamics; PA, photoacoustic; MAP, maximum amplitude projection; L_1_ and L_2_, immiscible liquids; y and y’, the height of L-L interface; R and R’, curvature; and F and F’, focal length. Reproduced from Ref. [63] with permission from The Royal Society of Chemistry.

Kim et al. proposed a dielectro-optofluidic lens (DOL) that manipulates the optical focal length by changing the lens-acting liquid level in the DOL using electrical signal [63]. The developed DOL consisted of three electrodes to force electrical signal and two immersible liquids: low-density electro-hydro-dynamics (EHD) driving liquid and high-density meniscus-forming liquid (Fig. 4(a)). By using the EHD-flow, generated by applying a non-uniform electric field to a dielectric liquid containing a small amount of polar surfactant, the height (y and y’) and curvature (R and R’) of the two liquids can be adjusted by controlling the focal length (F and F’) of the light passing through (Fig. 4(b)). They demonstrated the extended DoF by oscillating the focal length and the ability to maximize the PA signal by adjusting the focal length (Fig. 4(c)).

Another method to shift the optical focus is to use a tunable acoustic gradient (TAG) lens. The TAG lens is an adaptive lens that can change its effective focal length in a few microseconds or less [64], so it has been widely used for varifocal imaging [[65], [66], [67]]. Yang et al. in 2017 reported an OR-PAM system using a TAG lens to extend the DoF [68]. The TAG lens consisted of a cylindrical piezoelectric shell filled with silicone oil and its focal length can be shifted according to the applied sinusoidal signal. When the TAG lens was off, the lateral resolution of the system in a carbon fiber image was measured to be 3.3 μm and the FWHM was maintained at ˜8 μm for the depth range of about 160 μm. With the TAG lens on, the depth range extended to 750 μm with the other imaging parameters remaining the same. In the same year, they introduced another multifocus OR-PAM technique to save the axial scanning time [69]. They divided a single laser pulse into 3 pulses and applied different time delays through 3 multimode fibers of different lengths. As a result, they were able to acquire 3 B-mode images of a different focal depth simultaneously and expanded the DoF.

### 4.3 Propagation-invariant sinusoidal fringes (OR-PAM)

**Fig. 5:**
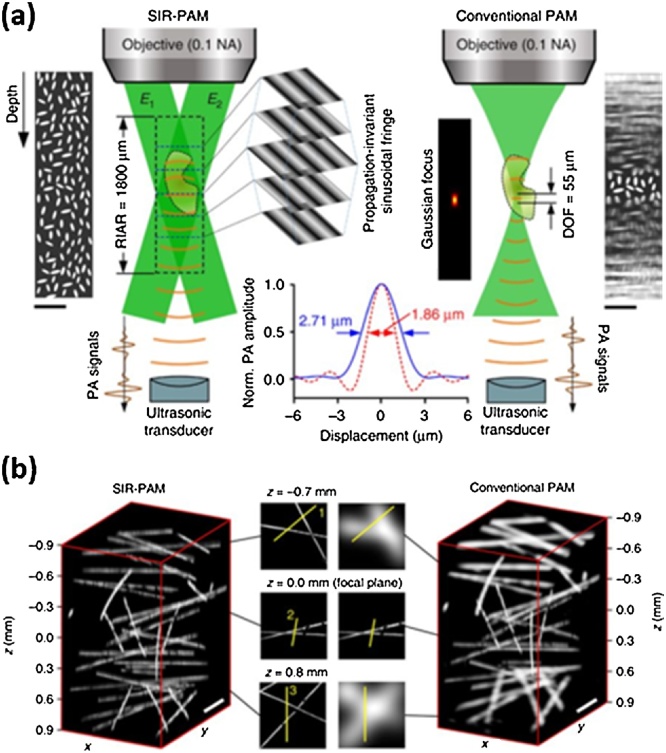
Motionless SIR-PAM vs. conventional OR-PAM. (a) The concept and the measured lateral resolution, RIAR, and DoF (b) Volume-rendered images of carbon fibers. PAM, photoacoustic microscopy; SIR-PAM, spatially invariant resolution PAM; OR-PAM, optical-resolution PAM; E1 and E2, symmetrical plane waves of the propagation-invariant sinusoidal fringes; RIAR, resolution-invariant axial range; and DoF, depth of field. Reproduced with permission from Ref. [70].

Yang et al. introduced an imaging technique using the single-pixel imaging method called motionless volumetric spatially invariant resolution PAM (SIR-PAM) [70] with the improved DoF and lateral resolution [71]. The single-pixel imaging method is an image reconstruction technique that uses a fixed single photodiode and light with the following sinusoidal patterns:

(5) $$P_{\varphi }\left(x,y,f_{x},f_{y}\right)=a+b∙cos\left(2\pi f_{x}x+2\pi f_{y}y+\varphi \right),$$

where (x,y) denotes the Cartesian coordinates, φ denotes phase shift, a denotes the averaged image intensity, b denotes the contrast, and $\left(f_{x},f_{y}\right)$ denotes the spatial frequency of the sinusoidal light pattern. To use this technique for 3D PA image reconstruction, Yang et al. used a US transducer instead of the photodiode and generated propagation-invariant sinusoidal fringes (PISFs) using a digital micro-mirror device (DMD) (Fig. 5(a)). The detected PA signal, $S_{\varphi }$, can be expressed as

(6) $$S_{\varphi }\left(f_{x},f_{y},z\right)=\int \int \mu (x,y,z)P_{\varphi }\left(x,y,f_{x},f_{y}\right)dxdy.$$

where z denotes the axial Cartesian coordinates and μ(x,y,z) is optical absorption distribution. Using various PISFs with four phase shift ($\varphi =0,\frac{\pi }{2},\pi ,\frac{3\pi }{4})$, the μ(x,y,z) can be calculated as follows

(7) $$\mu \left(x,y,z\right)=\frac{1}{2bk}F^{-1}\left[\left(S_{0}\left(f_{x},f_{y},z\right)-S_{\pi }\left(f_{x},f_{y},z\right)\right)+j\left(S_{\pi /2}\left(f_{x},f_{y},z\right)-S_{3\pi /4}\left(f_{x},f_{y},z\right)\right)\right]$$

where k is a constant depending on the detector sensitivity. Using this approach, the SIR-PAM showed the lateral resolution of 1.89 μm and the resolution-invariant axial range (RIAR) of 1800 μm, whereas the conventional OR-PAM sharing the same objective lens showed the lateral resolution of 2.86 μm and the DoF of 55 μm.

### 4.4 Synthetic aperture focusing technique (SAFT, AR-PAM)

**Fig. 6:**
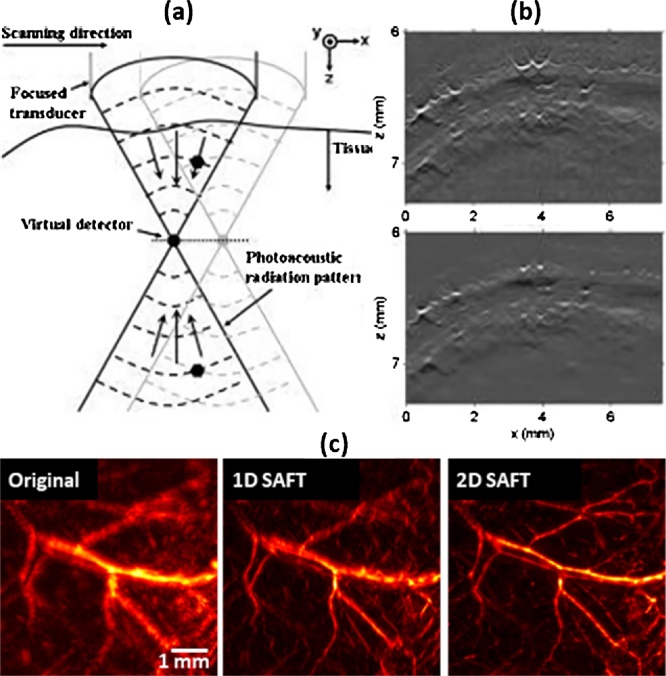
(a) Schematic of 1D SAFT with the virtual-detector concept. (b) Original (top) and 1D SAFT (bottom) AR-PAM B-scan image of a rat’s scalp. (c) Original (left), 1D SAFT (middle), and 2D SAFT (right) AR-PAM MAP image of mouse leg vessels. SAFT, synthetic aperture focusing technique; AR-PAM, acoustic-resolution photoacoustic microscopy; and MAP, maximum amplitude projection. Reproduced with permission from Ref. [72,76].

The AR-PAM also has the severely degraded lateral resolution and SNR in the out-of-focus region. The degradation of AR-PAM is due to the high acoustic NA rather than the optical focus. In the AR-PAM, the US transducer detects all signals generated within the acoustic NA. Thus, during the raster scanning, the same PA signals are repeatedly detected by the US transducer with different time delays, resulting in the distortion of the PA signals in the out-of-focus region. In 2006, to overcome this problem, Li et al. first introduced SAFT in AR-PAM using delay-and-sum (DAS) algorithm, which accumulates the corresponding PA signals originating from the same source thus correcting the distortion (Fig. 6(a, b)) [72]. Since then, Part et al. proposed a way to further improve the resolution of the 1D SAFT using the delay-multiply-and-sum algorithm (DMAS) [39].

Meanwhile, several studies have been conducted to extend the SAFT dimension by accumulating the PA signal in cross directions [73] or spherical directions [74]. However, the resolution enhancement has been limited due to the mismatch between the actual PA wavefronts and the estimated PA signal to be accumulated in the SAFT processes. This mismatch is due to a wrong assumption that blood vessels generate spherical ultrasound wavefronts. To address this problem, Deng et al. proposed an adaptive SAFT that applies additional 1D SAFT processes in the directions perpendicular to each vessel after the conventional 2D SAFT [75]. In 2017, Cai et al. proposed to apply 3D blind deconvolution to complement the previous SAFT methods. However, since the time delay of the original PA signal varies depending on the depth, it is difficult to estimate the 3D PSF. Alternately, they firstly corrected the distortion through the previous 2D SAFT method [74], so that the PSF becomes depth-independent. They then enhanced the image quality by applying the Richardson-Lucy deconvolution algorithm at each depth in the SAFT image. As a result, for the depth range of 1800 μm, the lateral resolution improved from ˜75 μm (with SAFT alone) to ˜29 μm when the deconvolution was applied to the 2D SAFT alone results. Note that, the lateral resolution was not optimal up to 700 μm in the original AR-PAM image. However, because these methods are based on the initial 2D SAFT with the wrong assumption, there was still a fundamental limitation on the final image quality.

In 2018, Jeon et al. presented a new 2D SAFT method that can solve the wavefront mismatch problem of the previous SAFT methods (Fig. 6(c)) [76]. Instead of accumulating all the corresponding signals, they performed independent 1D SAFT in multiple directions. The 1D SAFT in the Fourier domain enhances the high-frequency components of the PA image only in a certain direction and represents the best enhancement when the SAFT direction and sample direction are orthogonal. They accumulated the enhanced frequency components in the Fourier domain and performed inverse Fourier transform to reconstruct the 2D SAFT image with the improved lateral resolution in all directions. They demonstrated the superiority of the proposed 2D SAFT by quantitatively comparing the proposed 2D SAFT with the previous 2D SAFT methods.

## 5 Advances in scanning methods

Fast imaging speed is a crucial factor for high throughput studies. Since the optical microscopy systems use optical scanning mirrors, they typically have fast imaging speed. However, PAM systems cannot use conventional optical scanning mirrors directly for faster imaging speed since they need a scanner that can reflect both the excitation optical beam and the generated US waves. For this reason, many PAM systems are primarily based on a mechanical scanning method and are limited in speed. Voice coil is one of the fastest mechanical scanning methods [77,78], but its scanning speed is fundamentally limited by the driving force and the mass of the scanner head. In recent years, many scanning mirrors have been developed that can operate in water to overcome the speed limitations. Commonly, micro-electro-mechanical systems (MEMS) and galvanometer-based scanning methods are used for the scanning mirror systems. The MEMS and galvanometer scanners have miniaturization and stability advantages, respectively. Additionally, a polygon-mirror scanner with faster scanning speed has been introduced and we also reviewed the research at the end of this section.

### 5.1 Water immersible MEMS scanner

**Fig. 7:**
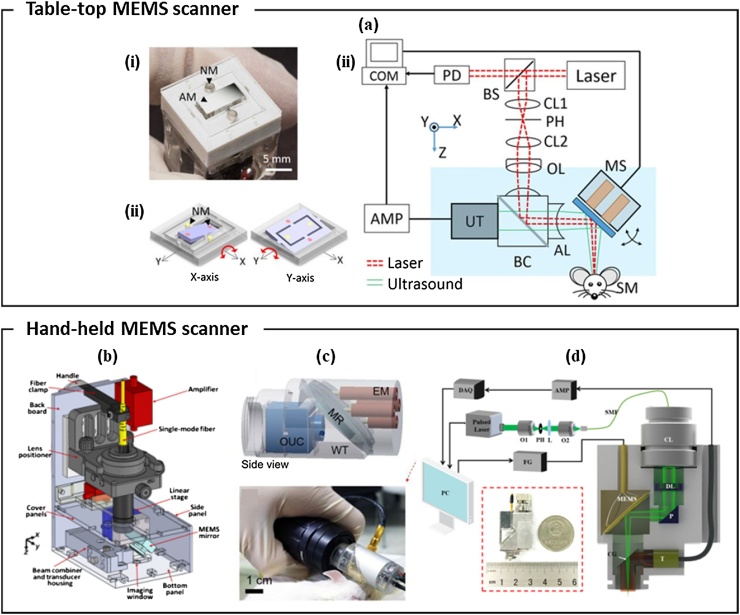
(a) (i) Photograph, (ii) scanning mirror schematic, and (iii) system setup of the 2-axis MEMS scanner OR-PAM. NM, neodymium magnet; AM, aluminum mirror; COM, computer; PD, photodiode; BS, beam splitter; AMP, amplifier; UT, ultrasound transducer; CL, condenser lens; PH, pinhole; OL, objective lens; BC, beam combiner; AL, acoustic lens; MS, MEMS scanner; and SM, sample; and CPL, carbon particles lump. (b) Handheld OR-PAM system using a 1-axis MEMS scanner. (c) Scanner schematic (top) and photograph (bottom) of handheld PAM with a 2-axis MEMS scanner. EM, electromagnets; MR, mirror; OUC, opto-ultrasound combiner; and WT, water tank. (d) Schematic of an ultra-compact portable PAM with a 2-axis MEMS scanner. O, objective lens; DAQ, data acquisition card; L, convex lens; SMF, single-mode fiber; CL, collimation lens; DL, doublet lens; P, prism; CG, cover glass; FG, function generator; T, transducer; MEMS, micro-electro-mechanical system; and OR-PAM, optical-resolution photoacoustic microscopy. Reproduced with permission from Ref. [[82], [83], [84], [85]].

In the early stages of PAM systems design, the optical scanning methods manipulated the light beam within the fixed acoustic focus to obtain the image [79,80]. This method had limited FOV and SNR. In 2012, Yao et al. reported a fast PAM system that overcame this issue by using an opto-acoustic combiner and a water-immersible MEMS scanner [81]. The excitation laser beam and the PA waves were coaxially aligned by the combiner and simultaneously steered by the MEMS scanner. They achieved high imaging speed of 400 Hz/B-mode and the high SNR of 36.5 dB. However, it was difficult to miniaturize the system because of the motorized linear stage used for volumetric scanning. In 2015, Kim et al. introduced an OR-PAM system with a 2-axis MEMS scanner (Fig. 7(a)) [82]. The 2-axis MEMS scanner was small (15 mm × 15 mm × 15 mm) and had wide scanning range (9 mm × 4 mm) and fast-imaging speed (50 Hz and 30 Hz in the fast and slow scanning direction, respectively). This compact 2D-axis MEMS scanner became the basis for the future hand-held PAM systems discussed below.

Since 2012, many research groups have tried to develop handheld-type OR-PAM systems using small size MEMS scanners to image various anatomical sites. Lin et al. reported a compact handheld OR-PAM based on a 1-axis MEMS scanner and a linear stage (Fig. 7(b)) [83]. The scanning range was 2.5 mm × 2.0 mm, and the scanning speed was 220 Hz in the lateral direction and 1 Hz in the elevational direction. Thus, they could image the human cuticle and mole at the scanning speed of 2 Hz in bidirectional scanning mode. However, the probe still was relatively large (80 mm × 115 mm × 150 mm) due to the linear stage and thus was limited in applications. Park et al. presented a smaller handheld PAM using a 2-axis MEMS scanner (Fig. 7(c)) [84]. Because the 2-axis MEMS scanner does not require the linear stage for volumetric scanning, they could fabricate more compact probe with a diameter of 17 mm, a weight of 162 g, and a maximum imaging range of 3 mm × 4 mm. With the handheld PAM system, they imaged blood vessels of mice ear, iris, and brain at an imaging speed of 35 Hz/B-scan. In 2018, Chen et al. reported an ultra-compact portable PAM using a 2-axis MEMS scanner and an unfocused US transducer (Fig. 7(d)) [85]. The system probe weighted only 20 g and was 22 mm × 30 mm × 13 mm in size. They imaged the rat abdominal cavity and the human oral cavity with a 2 mm × 2 mm FOV. The resonant frequency of the scanner was ˜800 Hz that could support up to 3.2 Hz imaging speed. Nonetheless, the volumetric imaging speed was limited to 0.2 Hz because of the laser repetition rate of 50 kHz and the SNR was limited because of the fixed unfocused US transducer.

### 5.2 Galvanometer scanner

A galvanometer scanner could also be a good alternative to the MEMS scanner due to its stability and fast speed. Yuan et al. first introduced an OR-PAM system using a 2D galvanometer scanner [86]. They successfully demonstrated *in vivo* vascular imaging and *ex vivo* erythrocyte imaging with the lateral resolution of ˜500 nm. However, real-time imaging could not be achieved due to the slow laser repetition rate of 15 Hz. In addition, both the FOV and the SNR in this system were severely limited by the unfocused US transducer.

**Fig. 8:**
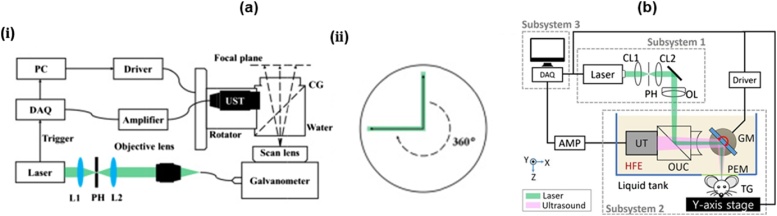
(a) (i) System schematic and (ii) scanning mechanism of the large-FOV OR-PAM using a 2D galvanometer scanner and a cylindrical focused US transducer. black arrows: optical scan line, green bar: acoustic focal zone, dotted arrow: rotational scanning trace; FOV, field of view; OR-PAM, optical-resolution photoacoustic microscopy; L, lens; UST, ultrasound transducer. (b) Schematic of the galvanometer scanner OR-PAM using a non-conducting liquid. GM, galvanometer; CL, condenser lens; PEM, polyethylene membrane; TG, target; and OR-PAM, optical-resolution photoacoustic microscopy. Reprinted with permission from Ref. [88,89].

To improve the imaging speed and the SNR, Jin et al. in 2017 presented a new galvanometer scanner-based PAM with a cylindrically focused US transducer [87]. Using the galvanometer scanner, they optically scanned the cylindrically focused area to achieve high SNR. Simultaneously, they rotated both US transducer and the optical scanning area together to obtain volume image data. It took 35 s to get a typical volumetric image data. The FOV was approximately limited to 7 mm in diameter. In 2018, Qin et al. upgraded the system developed by Jin et al (Fig. 8(a, i)) [88]. They moved the axis of rotation of the US transducer to one end of the cylindrical focus, in the process increasing the diameter of the FOV to 40 mm (Fig. 8(a, ii)). The typical imaging time remained similar to the previous system [87] because of the limited repetition rate of the laser.

The SNR is maximized when the acoustic waves are focused spherically and are aligned coaxially with the optical beam. However, this is difficult to achieve with the galvanometer since commercial galvanometers cannot operate in water. Kim et al. addressed this problem by using a non-conducting liquid (Novec 7500 Engineering Fluid, 3 M, USA) as an acoustic impedance matching medium instead of water [89]. In the non-conducting liquid, the system coaxially aligned the two beams via an opto-acoustic beam combiner and steered their foci (Fig. 8(b)). In this way, they could obtain the images with a high SNR of 44 dB and a wide FOV of 4 mm × 8 mm within 2 s. They also compared the acoustic attenuation in water and the non-conducting liquid and observed that there was higher attenuation in the non-conducting liquid.

### 5.3 Polygon-mirror scanner

**Fig. 9:**
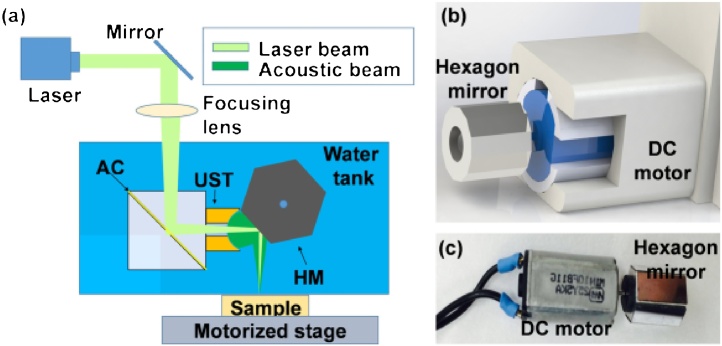
(a) Schematic of the OR-PAM system with a hexagon-mirror scanner (b) 3D rendered image and (c) photograph of the hexagon-mirror scanner. OR-PAM, optical-resolution photoacoustic microscopy; AC, aluminum coating; UST, ultrasound transducer; and HM, hexagonal mirror. Reproduced with permission from Ref. [90].

Lan et al. recently developed a high-speed OR-PAM with a hexagon-mirror scanner and demonstrated PAM imaging at speeds up to 900 Hz/B-scan (Fig. 9) [90]. The system scanned the sample using a waterproof DC motor that rotated the hexagonal mirror at a constant speed. Each facet of the hexagonal mirror was 5 mm × 8 mm and it was coated with aluminum to reflect the acoustic and optical beams simultaneously. Unlike MEMS and galvanometer scanners, this hexagonal-mirror scanner system does not require repetitive acceleration and deceleration, which allows for fast and reliable scanning. In particular, MEMS has a limited scanning speed because the stability of the scanning depends on the resonance frequency of the scanner. In addition, since the DC motor used in the scanner could operate in the water, it was possible to steer the coaxially aligned beams and consequently, a high SNR could be obtained. Besides, the waterproof scanner has the advantage of using water instead of non-conductive liquids with low acoustic impedance. However, 40% of the A-lines at the end of each B-scan cannot be used because the beam was blocked by the transducer or the step size and lateral resolution increased and only the remaining 60% of the A-lines could be used. This inefficiency might be improved by increasing the number of polygon faces.

### 5.4 Microlens array

**Fig. 10:**
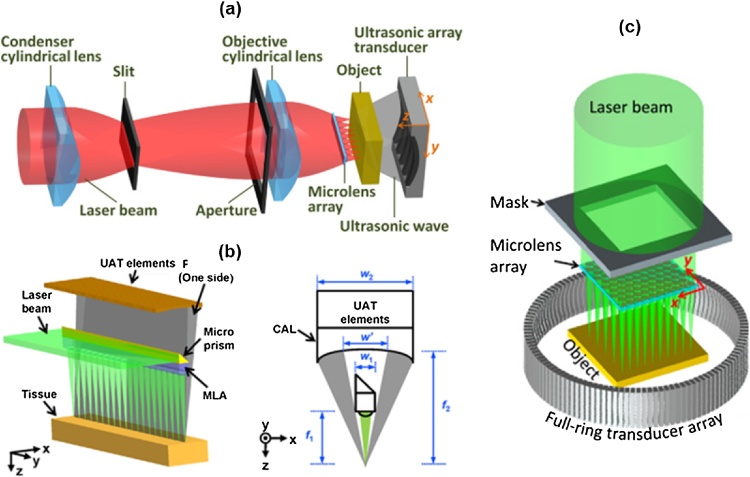
Schematics of (a) the transmission-mode multifocal OR-PAM system, (b) reflection-mode multifocal OR-PAM system, and (c) multifocal PA computed microscopy. OR-PAM, optical-resolution photoacoustic microscopy; and PA, photoacoustic. Reproduced with permission from Ref. [[91], [92], [93]].

Single ultrasound transducer-based scanning methods have a fundamentally limited speed because they must scan the region of interest point by point. Song et al. proposed a multifocus OR-PAM that uses a linear ultrasound array transducer with 48 elements instead of a single transducer to solve this problem (Fig. 10(a)) [91]. Array transducers can receive ultrasound in a large area and can reconstruct images in real time without mechanical scanning. They optically stimulated 20 spots at a time via a microlens array. They then scanned the sample in the lateral and elevational directions to obtain a volumetric image. As a result, they obtained a volumetric image with the size of 1000 × 500 × 200 voxels and the 10-μm lateral resolution within 4 min. However, this system was designed as a transmission-mode PAM system, so it was difficult to demonstrate *in vivo* imaging. For that reason, Li et al. developed a reflection-mode multifocus OR-PAM in which a microlens array is embedded underneath an array transducer (Fig. 10(b)) [92]. They placed a microprism at the top of the microlens to reflect laser beams vertically onto a sample. In this way, they imaged a mouse ear and brain with FOV of 6 mm × 5 mm and the lateral resolution of 16 μm within 2.5 min. The above both multifocal OR-PAM systems used an 8-channel data acquisition board and a 6:1 multiplexer. If they used a data acquisition board with more channels, the scan would have been faster. Xia et al. developed a multifocus PA computed microscopy system that uses a 2D microlens array and a ring array transducer (Fig. 10(c)) [93]. They transmitted 1800 optical foci to a sample through the 2D microlens array and raster-scanned at 25-μm intervals. They then digitized the PA signal with the 512-element ring array transducer and a 48-channel digitizer. As a result, they were able to obtain a cross-sectional image with a FOV of 10 mm × 10 mm and the lateral resolution of 29.4 μm. In this study, the image speed was limited by the digitizer operating at 5 Hz. Thus, they compromised between the image quality and speed, and eventually used 25% of the elements to acquire a cross-sectional PAM image within 36 s.

Table 1 summarizes the specifications of the fast-scanner-based PAM systems described above. We categorized the systems according to their portability, coaxiality, acoustic focus, and scanner types.

**Table 1:** Specification of fast-scanning PAM systems.

Ref.	Scanner	Portability	Coaxiality	Acoustic focus	Scanning speed	Imaging range	Lateral resolution
[77]	Voice coil	Table top	O	Spherical	20 Hz/B-scan 40 Hz/B-scan	9 mm 1 mm	3.4 μm
[81]	1D MEMS +Motor	Table top	O	Spherical	400 Hz/B-scan	3 mm	˜10 μm
[82]	2D MEMS	Table top	O	Spherical	50 Hz/B-scan	9 mm	3.6 μm
[83]	1D MEMS +Motor	Hand held	O	Spherical	220 Hz/B-scan	2.5 mm	5 μm
[84]	2D MEMS	Hand held	O	Spherical	35 Hz/B-scan	2.8 mm	12 μm
[85]	2D MEMS	Hand held	X	Unfocused	5 sec/volumetric scan	2 × 2 mm^2^	3.8 μm
[87]	Galvanometer	Hand held	X	Cylindrical	35 sec/volumetric scan	3.5^2^ × π mm^2^	10.4 μm
[88]	Galvanometer	Hand held	X	Cylindrical	34 sec/volumetric scan	20^2^ × π mm^2^	11.2 μm
[89]	Galvanometer	Table top	O	Spherical	2 sec/volumetric scan	4 × 8 mm^2^	6 μm
[90]	Hexagon mirror	Table top	O	Spherical	900 Hz/B-scan	9 mm	10 μm
[92]	Microlens + Motor	Table top	O	Linear array	2.5 min/volumetric scan	6 × 5 mm^2^	16 μm
[93]	Microlens + Motor	Table top	X	Ring array	36 sec/cross-sectional scan	10 × 10 mm^2^	29.4 μm

## 6 Advances in all-optical detection

The piezoelectric ultrasonic transducer is the most commonly used sensor to detect acoustic pressure in PAM. Today, many transducers of various sizes, frequencies, and focal lengths are commercially available, so users can choose the right specifications for their application. However, transducers are opaque and require coupling media, which inevitably complicates the PAM system design and makes its performance sub-optimal. To solve this problem, there have been many attempts to replace the acoustic detection method with an all-optical detection method. We will review two representative technologies for all-optical PAM (AOPAM): Fabry-Perot (FP) sensor and remote sensor.

### 6.1 Fabry-Perot sensor

In 2008, Zhang et al. developed AOPAM using a FP interferometer [94]. The FP interferometer consisted of a wedged polymethylmethacrylate (PMMA) and a polymer (parylene C) film spacer. The polymer film was sandwiched by two dielectric dichroic mirrors that reflect light at wavelengths between 1500 nm and 1650 nm and transmit light at wavelengths between 600 nm and 1200 nm. They excited the fixed wide area using a pulsed laser with a transparent wavelength in the mirrors. At the same time, they scanned the FP sensor with a continuous-wave (CW) 1550-nm interrogation beam using a galvanometer scanner. Simultaneously, the incident irradiation beam is reflected by the two mirrors and detected by a photodiode. Its reflectivity changes sensitively to the interrogation beam wavelength and thickness of the polymer spacer. Accordingly, the reflectivity can be modulated when ultrasound pressure changes the film thickness.

Thus, by detecting reflected interrogation beam power, the acoustic pressure can be detected. This approach uses a purely optical scanning method. Hence, it can avoid the slow mechanical scanning or the complex optical design to bypass the transducer in existing PAM systems. In addition, FP sensors have a wide bandwidth, especially at a DC frequency, compared to conventional transducers. Therefore, the FP sensor can sensitively detect the PA signal even from large absorbers. They have successfully demonstrated *in vivo* all-optical imaging of blood vessels in human palms, mouse skin [95], and mouse embryos [96] using this FB interferometer-based PAM.

However, there was a slow scanning speed problem. As described, FP interferometer-based PAM illuminates a wide field, requiring a high-power excitation laser source. However, many commercial high-power lasers have a slow PRF, and this slow PRF (50 Hz in [96]) results in a bottleneck in imaging speed. To overcome this problem, Huynh et al. proposed ways to accelerate the detection speed by parallelizing the detection through multiple interrogation beams [97,98] and by reconstructing the images with sparse data via a compressed sensing technique [99]. As a result, they achieved a fast scan rate of ˜10 s at a FOV of 10 mm *×* 10 mm using a 200-Hz pulsed laser.

**Fig. 11:**
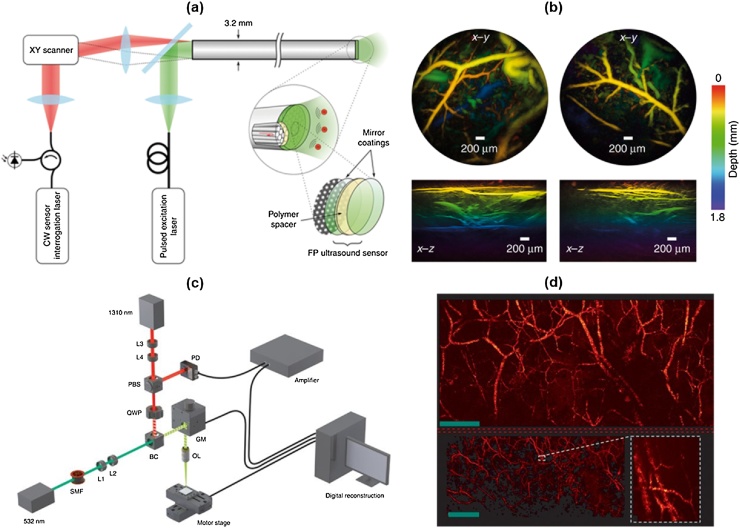
(a) System schematic of all-optical forward-viewing PA endoscopy probe. (b) Mouse abdominal image acquired by the all-optical PA endoscopy probe. (c) Experimental setup of PARS microscopy. (d) Mouse ear image acquired by the PARS microscope. PA, photoacoustic; and PARS, PA remote sensing. Reprinted from Ref. [101,109]. © The Authors, some rights reserved; exclusive licensee American Association for the Advancement of Science. Distributed under a Creative Commons Attribution Non Commercial License 4.0 (CC BY-NC) http://creativecommons.org/licenses/by-nc/4.0/.

This all-optical PAM method has been also developed as a handheld probe type. In 2017, Ansari et al. introduced an all-optical forward-viewing PA endoscopic probe [100]. They delivered the interrogation beam through a fiber bundle consisting of 18,000 cores and scanned the FP detector over the bundle by scanning the proximal end of the bundle. The inner diameter of the bundle was 1.25 mm, but the imaging aperture was expanded to a diameter of 5 mm using a free-space 1:4 optical relay. In the study, the measured -3 dB bandwidth was 68 MHz and the lateral and the axial resolution were 57 μm and 46 μm, respectively, at 1-mm depth. In 2018, they developed a miniaturized all-optical PA endoscopic probe with a diameter of 3.2 mm (Fig. 11(a and b)) [101]. Instead of scanning the FB sensor in free space, they deposited the FP on the distal side of a bundle with 50,000 12-μm fiber cores. The lateral and the axial resolution were 39 μm and 31 μm at 1-mm depth, respectively.

### 6.2 Remote sensor

In addition to the problems associated with the use of the ultrasound transducer described above, there has been a problem that PAM requires coupling media, such as water or ultrasound gels. This problem can be a barrier to many clinical and preclinical applications. For this reason, many non-contact PAM techniques have been actively studied. Initially, the main approach for remote sensing was to use an optical interferometer [[102], [103], [104], [105], [106], [107]]. If a PA signal is generated below the skin after laser excitation, the acoustic pressure induces surface displacement. Because the local acoustic pressure is proportional to the velocity of the displacement [108], the initial pressure image can be predicted by measuring the surface displacement with the interferometer. However, these methods have a limitation that the interferometry sensor is sensitive to unwanted phase and reflectivity modulation.

In 2017, Hajireza et al. introduced non-interferometric PA remote sensing (PARS) microscopy (Fig. 11(c and d)) [109]. Analogously to the previous remote sensing PAM techniques, PARS also employs modulation of a reflected CW interrogation beam. However, PARS does not use an interferometer, so it is not sensitive to the phase oscillation and exclusively detects the intensity modulation. In addition, PARS reads the intensity reflectance at the photoacoustic source under the skin surface. This feature is distinguished from conventional OR-PAM and remote sensing PAM, which measure propagated ultrasonic pressure on the surface. Hajireza et al. have experimentally demonstrated this feature by showing that there is little time delay between the excitation pulse and the detected PA signal. As a result, they demonstrated *in vivo* remote PAM imaging of the mouse ear with a high SNR of ˜40 dB using excitation laser energy of ˜ 40 nJ/pulse and 4-mW interrogation laser power. The lateral and the axial resolution were measured as 2.7 μm and 43 μm, respectively. In 2018, they developed a new remote PAM technique with a deeper imaging depth, called deep PARS (dPARS) microscopy [110]. In the new dPARS system, the photodiode of the previous PARS system was replaced by a balanced photodiode to improve the SNR. They also extended the maximum imaging depth using the NIR interrogation beam that has a longer mean-free path than the excitation beam. Theoretically, the mean-free paths in soft tissue for 532 nm and 1310 nm light are 0.57 mm and 1.43 mm, respectively. In carbon network imaging, the lateral and the axial resolution were measured as ˜7 μm and about 30 μm, respectively. They then demonstrated *in vivo* mouse ear imaging with an imaging depth of 1.2 mm and an SNR of ˜ 50 dB using excitation laser power of ˜60 nJ/pulse and interrogation laser power of 5 mW.

## 7 Preclinical and clinical applications

In this section, we describe the representative PAM applications. In preclinical applications, PAM has been widely used in the research fields of neuroscience and cancer biology for microangiography, functional imaging, and molecular information. In clinical applications, it is used for histology and dermatology studies.

### 7.1 Microangiography in cancer biology and neuroscience

**Fig. 12:**
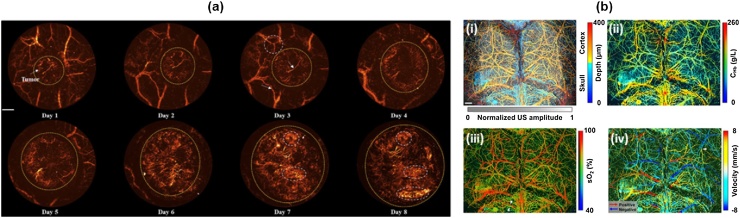
(a) *in vivo* PAM monitoring of mouse LS174 T tumor angiogenesis. Scale bar: 1 mm. (b) Multi-parametric PAM images of a mouse brain with an intact skull. (i) depth-encoded skull and cortical vasculatures, (ii) total concentration of hemoglobin, (iii) oxygen saturation, (iv) blood flow. Scale bar: 0.5 mm. PAM, photoacoustic microscopy. Reproduced with permission from Ref. [87,112].

It is a well-known fact that a great deal of neovascularization takes place to supply nutrients to the developing cancer cells. Thus, monitoring the angiogenesis with PAM may potentially lead to an early cancer screening tool in clinical applications. Jin et al. demonstrated the angiogenesis monitoring of growing tumor and visualization of human oral vessel networks using a portable OR-PAM [87]. They also monitored the angiogenesis in mouse LS174 T tumor (Fig. 12(a)). As per their observation, as the tumor grows, the newborn vessels spread out around the tumor region. The PA imaging was able to monitor the cancer growth process and environmental changes needed for early cancer diagnosis. In addition, blood vessel networks in human tongue and lips were also acquired with the portable PAM system, confirming the great potential of PAM for screening early cancers in humans.

PAM can also monitor brain hemodynamic activities by imaging small blood vessels. Conventionally, functional magnetic resonance imaging (fMRI), two-photon microscopy, wide-field optical microscopy are widely used for the brain hemodynamic studies. However, these methods suffer from several disadvantages including poor spatial resolution, shallow imaging depth, and the use of external contrast agents. In contrast, PAM addresses these problems and measures multiple functional parameters such as sO2, cerebral blood flow (CBF), flow speed, oxygen extraction fraction (OEF), and cerebral metabolic rate of oxygen (CMRO_2_) (Fig. 12(b)) [111,112]. In 2015, Yao et al. demonstrated fast functional OR-PAM imaging of the whole mouse brain with an intact skull using a 1D MEMS scanner [113]. They quantified CBF, OEF, and CMRO_2_ of each cerebral vessel, and observed their changes induced by electrical stimulations on the mouse hindlimbs. They also succeeded in distinguishing the activated somatosensory regions by stimulating the left and the right hindlimbs separately.

### 7.2 Contrast-enhanced imaging

**Fig. 13:**
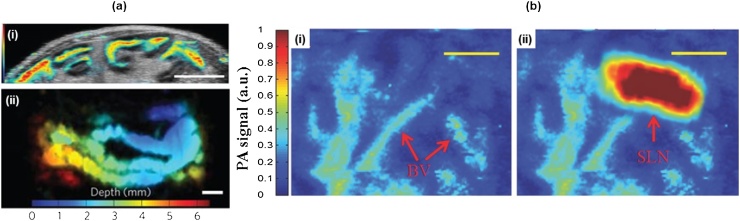
(a) AR-PAM (color) and ultrasound (grey) B mode image (i) and depth-encoded AR-PAM MAP image (ii) of mouse intestine after injection of ZnBNc nanonaps. Scale bar: 5 mm (b) AR-PAM MAP images of SLN before (i) and after (ii) injection of N4 NPs. Scale bar: 1 mm. AR-PAM, acoustic-resolution photoacoustic microscopy; MAP, maximum amplitude projection; SLN, sentinel lymph node; and NP, nanoparticle. Reproduced with permission from Ref. [16,119].

PAM has great potential in molecular imaging because it is a non-ionizing and has a good spatial resolution. For example, PAM has monitored the transdermal delivery and diffusion processes of gold nanoparticles in blood capillaries by exploiting these advantages [114]. Recently, various contrast agents have been developed that are safe and provide a high optical absorption coefficient at specific wavelengths. NIR light can deeply penetrate into biological tissue. Thus, by using a contrast agent with a high absorption coefficient in this NIR range, PAM can image deep organs [115], such as a gastrointestinal (GI) tract (Fig. 13(a)) [16] and bladder [116]. For these applications, the dark-field AR-PAM is often used to maximize the imaging depth.

Sentinel lymph node (SLN) imaging could also become an important application of PAM. SNL biopsy (SLNB) is an essential diagnostic procedure for staging cancer. For effective SLNB, first, identifying the target SLNs is very important. The existing SLN imaging methods have several issues including ionizing radiation and low spatial resolution of the Geiger counter. Thus, many PA SLN imaging studies have been conducted to take advantages of the PA features to identify SLNs [14,117]. In 2015, Lee et al. introduced dual color SLN imaging using nanonaps [118]. The Nanonap is a newly reported organic dye with strong NIR absorption, multi-color capability, high spectral stability, and with no potential heavy metal toxicity [16]. They used dark-field AR-PAM with multiple wavelengths of 707 nm and 860 nm and imaged rat’s SLNs and lymphatic vessels *in vivo*. They could clearly distinguish the SLNs at 6 mm depth. In 2016, Cai et al. also showed PA molecular imaging of mouse’s SLNs using dark-field AR-PAM and gold nanoparticles (NPs) (Fig. 13(b)) [119]. In addition, they also demonstrated photothermal therapy using the functionalized NPs that can target breast cancer cells.

PAM can also be used to identify or treat the cancer cell using exogenous contrast agents [120,121]. Zhong et al. demonstrated tumor-targeting molecular imaging and therapy using nanoparticles, called PTX-PAnP [122]. The PTX-PAnP can be selectively bound with folate receptors that are overexpressed by human epithelial carcinoma cells (HeLa). The nanoparticles have an absorption peak at 770 nm and they release therapeutic drugs when irradiated by a pulsed laser. At the same time, they are visible in the PAM imaging to study the effectiveness of the treatment.

### 7.3 Histological examination

**Fig. 14:**
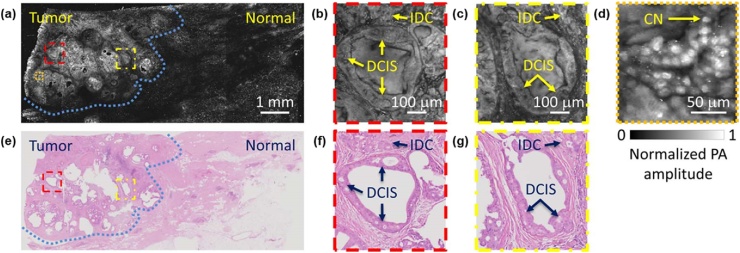
(a) UV-PAM image of a fixed breast tumor specimen. (b–d) Magnified images of the areas highlighted with dashed boxes in (a) (e) H&E stained image obtained by optical microscopy. (f–g) Magnified image of the areas highlighted with dashed boxes in (e). The areas highlighted with the blue dashed lines in (a, e) represent tumor regions. IDC, invasive ductal carcinoma; DCIS, ductal carcinoma in situ; CN, cell nuclei; and PA, photoacoustic. Reprinted from Ref. [23]. © The Authors, some rights reserved; exclusive licensee American Association for the Advancement of Science. Distributed under a Creative Commons Attribution Non Commercial License 4.0 (CC BY-NC) http://creativecommons.org/licenses/by-nc/4.0/.

The goal of breast-conserving surgery (BCS) is to minimize the risk of recurrence and cosmetic side effects. Intraoperative frozen section analysis (FSA) is known to be effective in achieving this goal because it allows immediate resection of residual disease [123]. However, FSA involves complicated procedures such as freezing, sectioning, and labeling. Moreover, it is also difficult to freeze and interpret the adipose-rich breast tissue, especially in BCS [124]. In contrast, PAM with an ultra-violet (UV) wavelength (UV-PAM) could image the cell nuclei without the complicated procedures because DNA and RNA in cell nuclei have a high absorption coefficient at the UV wavelength. Therefore, the use of UV-PAM can shorten the preparation time and improve the interpretation accuracy for the fat-rich samples. In 2017, Wong et al. demonstrated label-free UV-PAM imaging of formalin-fixed breast tissue (Fig. 14(a–d)) [23]. The UV-PAM image clearly showed the morphology of individual cell nuclei with the high resolution of 330 nm and differentiated the tumor region (blue dashed line in Fig. 14(a, e)). In the tumor region, ductal carcinoma in situ (DCIS) and invasive ductal carcinoma (IDC) were also observed. The UV-PAM images had a good correlation with the corresponding H&E stained images (Fig. 14(e–g)). However, the scanning speed of conventional OR-PAM systems, including UV-PAM, was quite slow (˜100 min to image 5 mm × 5 mm FOV in this study). The slow-speed problem can be solved by using MEMS [81], galvanometer scanner [86], or microlens array [125] as discussed before. In addition, the preparation speed can also be saved by combining the PAM system with auto specimen sectioning system [126]. Another problem of OR- or UV-PAM systems in this application is the poor axial resolution. As described above, the axial resolution is acoustically determined, so it might not be sufficient for optical sectioning in cell imaging. For this reason, it is recommended to slice the tissue as thin as possible. Incidentally, unlike other VIS and NIR rays, UV light can cause safety problems, such as creating carcinogens in living tissue or destroying connective tissue and collagen beneath the skin. Therefore, special attention should be paid to experimental safety in this application using a UV laser.

### 7.4 Dermatology

Dermatology is another potential clinical application for PAM. Conventionally, dermoscopy and human vision are used to diagnose skin diseases such as skin tumors, warts, or fungal infections. However, such inspection methods have disadvantages of shallow imaging depth and low optical contrast. PAM has deep imaging depth and high optical contrast to image skin chromophores (e.g., melanin). Moreover, the high sensitivity to hemoglobin is a great advantage in dermatology since the skin vasculature is a hallmark of the skin condition. For these reasons, recently, PAM is emerging as a new dermoscopy system.

**Fig. 15:**
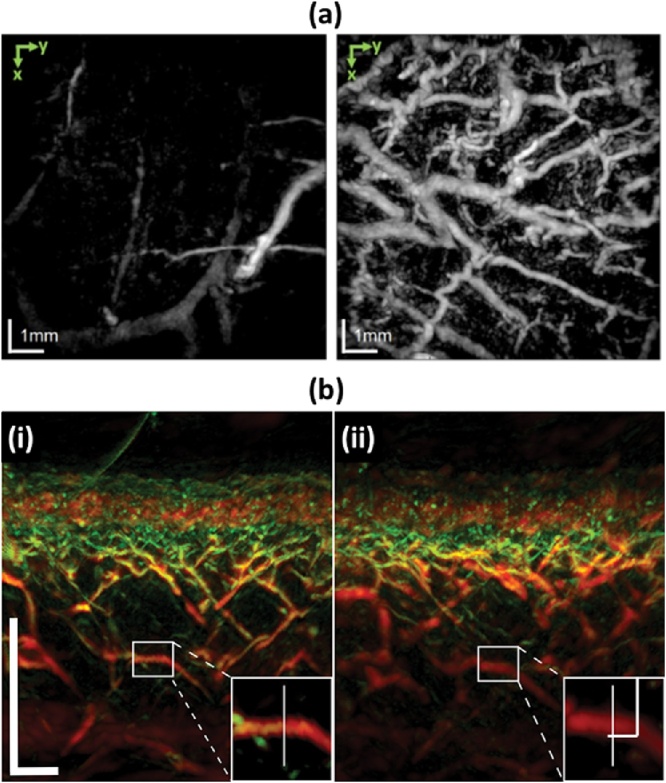
(a) PA MAP images of healthy skin (left) and diseased skin with chronic hyperkeratotic hand eczema (right). The projected depth range is from 1.5 mm to 2.5 mm. Scale bar: 1 mm. (b) PA B-mode images of forearm skin before (i) and after (ii) locally heating the surface. Scale bar: 500 μm. The images are made by combining two images obtained by a low-frequency US transducer (red) and high-frequency US transducer (green). Scale bar: 200 μm. Reproduced with permission from Ref. [127,129].

In 2015, Zabihian et al. demonstrated PA imaging of human skin [127]. The microvessel structures in the skin were imaged up to a depth of approximately 4 mm and visualized separately for each skin layer, such as papillary dermis, superficial vascular plexus, and subcutaneous tissue. They imaged the skin of the patients with atopic dermatitis, hyperkeratotic hand eczema, dyshidrotic hand eczema and then compared them with the PA images of the healthy skin. In the diseased skin images, they observed abnormalities like increases or decrease of vascularization, or vascular irregularities (Fig. 15(a)). In 2017, Aguirre et al. developed an ultra-broadband raster scan optoacoustic mesoscopy system (UB-RSOM) for skin imaging [128]. With the system, they successfully visualized the vascular patterns of the psoriatic and healthy skin and observed that vascularization in the dermis of the psoriatic skin was more active than in the healthy skin. In addition, using the same UB-RSOM system, Andrei et al. investigated the hyperemic response after locally heating the human forearm skin (Fig. 15(b)) [129]. The reactivity of the skin evaluated by the PA imaging system is expected to be useful for understanding the biology and physiology of the skin.

## 8 Conclusions and future perspectives

In this review, we introduced recent technical advances and the clinical and preclinical applicability of PAM. PAM has been developed over the years to have a better spatial resolution, higher SNR, faster imaging speed, and smaller size. In addition, PAM can be combined with other modalities [3] to observe more information other than the optical absorbance. Combined with pure optical modalities such as OCT [[130], [131], [132]] or fluorescence microscopy [41,133,134], PAM could analyze the various properties of a sample. As a result, the applications of PAM would be more diverse and wide.

However, PAM still faces several challenges. First, it is still difficult to display wide-field images in real time because most PAM systems acquire one dimensional depth-resolved PA signals per one laser pulse and reconstruct PA images from multiple scans. This disadvantage not only limits research throughput but also increases diagnosing time in clinical applications. The second problem is the SNR degradation at deep depth due to the optical and acoustic attenuation. The low SNR at deep depth can hinder full understanding of the underlying conditions of the relatively large tissue. Third, most PAMs require a coupling medium such as water or US gel, which can limit many intraoperative applications [135]. Many researchers are already exploring ways to overcome these problems using arrayed US transducer [136], fast PRF laser [137], compressed sensing [99], ultra-broadband detector [128], and non-contact detection [106,138]. In spite of these challenges, PAM has tremendously impacted the life and medical science researches. With further advances in technical development, PAM will become a more attractive biomedical imaging tool in the near future.

## Declaration of Competing Interest

None.
